# Engineered NF-κB siRNA-encapsulating exosomes as a modality for therapy of skin lesions

**DOI:** 10.3389/fimmu.2023.1109381

**Published:** 2023-02-08

**Authors:** Wei Lu, Jinzhong Zhang, Yungang Wu, Wenxue Sun, Zipei Jiang, Xu Luo

**Affiliations:** ^1^ The Quzhou Affiliated Hospital of Wenzhou Medical University, Quzhou People’s Hospital, Quzhou, Zhejiang, China; ^2^ Department of the Orthopedics of Traditional Chinese Medicine (TCM), the First Affiliated Hospital of Wenzhou Medical University, Wenzhou, Zhejiang, China; ^3^ Hemodialysis Room, Department of Nephrology, the First Hospital Affiliated of Wenzhou Medical University, Wenzhou, Zhejiang, China; ^4^ Department of Ophthalmology, the First Hospital Affiliated of Wenzhou Medical University, Wenzhou, Zhejiang, China; ^5^ Wenzhou Medical University, Wenzhou, Zhejiang, China; ^6^ Department of Wounds and Burns, The First Affiliated Hospital of Wenzhou Medical University, Wenzhou, Zhejiang, China; ^7^ Key Laboratory of Intelligent Treatment and Life Support for Critical Diseases of Zhejiang Province, The First Affiliated Hospital of Wenzhou Medical University, Wenzhou, Zhejiang, China; ^8^ Zhejiang Engineering Research Center for Hospital Emergency and Process Digitization, Wenzhou, Zhejiang, China

**Keywords:** engineered, siRNA, exosomes, UV, skin lesions, therapy

## Abstract

**Introduction:**

Despite the protection and management of skin has been paid more and more attention, effective countermeasures are still lacking for patients suffering from UV or chemotherapy with damaged skin. Recently, gene therapy by small interfering RNA (siRNA) has emerged as a new therapeutic strategy for skin lesions. However, siRNA therapy has not been applied to skin therapy due to lack of effective delivery vector.

**Methods:**

Here, we develop a synthetic biology strategy that integrates the exosomes with artificial genetic circuits to reprogram the adipose mesenchymal stem cell to express and assemble siRNAs into exosomes and facilitate in vivo delivery siRNAs for therapy of mouse models of skin lesions.

**Results:**

Particularly, siRNA enriched exosomes (si-ADMSC-EXOs) could be directly taken up by the skin cells to inhibit the expression of skin injury related genes. When mice with skin lesions were smeared with si-ADMSC-EXOs, the repair of lesioned skin became faster and the expression of inflammatory cytokines were decreased.

**Discussion:**

Overall, this study establishes a feasible therapeutic strategy for skin injury, which may offer an alternative to conventional biological therapies requiring two or more independent compounds.

## Introduction

Skin is the largest area organ in the human body, which has various functions such as regulating body temperature, preventing water loss, resisting the invasion of pathogens, and preventing body trauma (1, 2). In recent years, increased extreme weather and flourished means of chemoradiotherapy have damaged the skin by UV light and chemoradiotherapy drugs. The study confirmed that the skin of chemoradiotherapy patients, newborns, elderly people and long-term outdoor workers were more vulnerable (2, 3).

Chemotherapeutic drug extravasation is a common skin injury event in the clinic, however care guidelines developed for it can only alleviate the skin injury process in the manner of extrinsic intervention (4–7). UV light and radiation can cause irreversible damage to the skin such as photosensitivity and hyperpigmentation, and current anti-wrinkle, moisturizing, and whitening products only have reparative effects on the epidermal and dermal layers (8–10). Studies have confirmed that inhibiting the expression of key genes of skin lesions such as NF-κB, AP-1, and MMPs can improve cutaneous photosensitivity and dark pigmentation after chemotherapy (11, 12). Therefore, finding novel functional molecules that improve the body’s skin self-healing ability is a boon for patients with UV and chemotherapy skin lesions.

Exosomes are double membrane structured extracellular vesicles with diameters in the range of 30-200 nm (13). The special structure of exosomes can protect internal molecules from degradation by enzymes and stress, while having the ability of intercellular communication, and thus are commonly used as carriers for small molecule drug delivery (14). The variety and quantity of “cargo” loaded by natural exosomes are difficult to control, and only modified engineered exosomes can have the ability to selectively package nucleic acid drugs that target a gene of interest. Small interfering RNA (siRNA) is a short strand RNA targeting the 3’UTR region of mRNA, and siRNA can be loaded in exosomes across cells leading to silencing of their target genes (14, 15). The researchers constructed siRNA spherical nano-nucleic acids by the method of click chemistry, which can perform gene regulation and therapy on a psoriasis mouse model (15). Adipose derived mesenchymal stem cells (ADMSCs) are less difficult to access than umbilical cord and bone marrow mesenchymal stem cells, and exosomes (ADMSC-EXOs) produced by them can promote endothelial cell survival and repair dermal cells (16–18). Therefore, using ADMSCs as a carrier to biosynthesize engineered ADMSC-EXOs loaded with target siRNAs may be a promising novel gene therapy for repairing skin lesions.

Here, we *in vitro* packaged lentivirus containing siRNA against key genes (NF-κB) in skin lesions, and constructed stable cell lines secreting exosomes with high levels of siRNA (si-ADMSC-EXOs) by lentiviral transfection of ADMSCs. Subsequently, the knockdown effects of the engineered si-ADMSC-EXOs on the target genes were validated in *in vitro* cell experiments, and the therapeutic effects of the engineered si-ADMSC-EXOs on UVB induced skin lesions were further evaluated *in vivo* experiments in mice. In conclusion, we demonstrated its reparative effects on skin lesions *in vivo* and *in vitro* experiments by synthesizing engineered ADMSC-EXOs containing siRNA.

## Materials and methods

### Materials

C57BL6 males were purchased from the model animal Institute of Nanjing Medical University (Nanjing, China). Adipose mesenchymal stem cells (ADMSCs), HEK 293T and RAW 264.7 cell were purchased from ATCC cell bank (Shanghai, China). The synthetic siRNA was purchased from GenScript (Nanjing, China), and serum, DMEM medium was purchased from Thermo Fisher (USA). CD63, CD9, Calnexin and ALIX antibodies were purchased from Abcam (USA).

### Culture and engineering of ADMSC

Cells were cultured using DMEM medium containing 10% FBS. Lentiviral packaging plasmids containing siRNA and GFP fluorescent sequences, as well as lentiviral vectors containing puromycin, were transfected into logarithmically growing HEK 293T cells, changed 6 h later, and lentiviral supernatant 48 h after transfection was collected. Cells and cell debris were removed, and 50000 g was ultracentrifuged for 2 h to concentrate viral particles, which were resuspended using sterile PBS. At ADMSC cell density of 80%, approximately 10^7^ viral particles were added to 10^6^ cells for transfection. After 3 days, 10^-3^ of puromycin was added and siRNA containing stable ADMSC cells (si-ADMSCs) were selected.

### Cell proliferation assay

CCK-8 assay was performed to analyze cell proliferation and viability. A density of 2×10^3^ cells/well was seeded in 96 well plates. 10 μL of CCK-8 solution was added per well at 12, 24, 36, 48 and 60 (h), respectively, and then cell proliferation was determined at a wavelength of 450 nm using a microplate reader.

### Identification of exosomes

When cells reached 80% confluence, 10% exosome free FBS DMEM was used for rehydration, and cell supernatants were collected after 48 h of culture. Next, exosomes were obtained by centrifugation at 300 g for 10 min, followed by centrifugation at 10000 g for 1 h and ultracentrifugation at 110000 g for 70 min and resuspension with appropriate amounts of PBS. To know the number of exosomes, their particle size distribution and concentration were detected using a nanoparticle size tracking analyzer (NTA). The morphology of exosomes was observed using transmission electron microscopy (TEM). 30 μg of exosomal protein was used to perform Western blot (WB) analysis.

### Fluorescent tracing of MSC exosomes

Samples of freshly ultradissociated exosomes were diluted to 1 mL with diluent C and loaded with 6 μL PKH26 to the tube, mix gently for 30 s, let stand at room temperature for 5min. The reaction was quenched by adding 10% BSA in 2 mL PBS configuration and brought to 8.5 mL with serum-free medium. 1.5 mL of sucrose solution (0.971 M) was slowly added dropwise with a gun to an ultracentrifuge tube, and the exosome-PKH26 labeling solution was added above the sucrose buffer layer. 110000 g for over dissociation 2 h at 4 °C. The medium and middle layer were carefully aspirated and exosomes were resuspended with 1x PBS and transferred inside 10 kDa MWCO ultrafiltration tubes. Add 9 mL PBS, 0.75 mL culture medium, and centrifuge at 3000 g for 40 min to a final volume of 0.5-1 mL. The stained exosomes were added dropwise to epithelial cells cultured in serum-free exosomes, and the cells were collected after 4 h of culture for observation under a fluorescence microscope. Meanwhile, the stained exosomes were applied on the surface of mouse skin, and the absorption of exosomes was photographed under a fluorescence imaging system after 4 h.

### RT-qPCR

Total RNA from cells and tissues was extracted using Trizol as previously described (19). Reverse transcription and quantitative PCR primers for siRNAs were designed using miRNA design v1.01, and all primers were synthesized at GenScript Biotech (Nanjing, China). Briefly, 2 μL of total RNA was first mixed with 5 × gDNA wiper mix and incubated at 42°C for 2 min to remove contamination of genomic DNA. Incubate the mixture at 25°C for 5 min, 50°C for 15 min, and 85°C for 5 min. Finally, SYBR dye qPCR amplification was carried out on LightCyler 96 instrument. PCR program for tsRNAs included the following steps: 95°C for 10 min, followed by 40 cycles at 95°C for 15 s and 60°C for 1 min. The threshold cycle (CT) values were determined using a fixed threshold setting.

### Western blot

Exosomes or tissues were homogenized by protease free Congo beads, protein lysates were prepared using an ice bath of Pierce RIPA buffer containing protease and phosphatase inhibitors for 30min, and samples were quantified using a BCA protein assay kit. Protein samples were denatured by addition of SDS at 99° C for 5 min and run at 80 V for 30 min on 12% bis-tris stacking gel and 120 V for 1 h on separating gel according to the manufacturer’s recommended protocol. Proteins were transferred to 0.2 µm PVDF membranes. Membranes were blocked with 5% nonfat milk for 1 h at room temperature, incubated with antibodies against CD63, anti-CD9 as well as anti-AlIX in 1 x TBST overnight at 4°C, washed, and incubated with the corresponding secondary antibodies for 1 h at room temperature to detect target proteins. Finally, ECL luminescent liquid was added dropwise to expose in an exposure instrument.

### Animals and treatment conditions

All animal experiments were approved by the Laboratory Animal Ethics Committee of Wenzhou Medical University. 8 weeks old male C57BL/6 mice were randomly divided into four groups (n = 5), and an area of 3 × 3 cm^2^ just above the back of the mice was applied with hair removal cream, scraped off after 10 min, and 0.1 mg carprofen was administered to each mouse *via* drinking water. After 48 h, the UVB lamp height was set to 1 m (W = 10^7^ μJ/cm^2^), the mice in the 4 groups were irradiated for 0, 16, 32, and 64 (min) times per week until the mice developed macroscopic skin lesions in the skin. Twenty 8-week-old C57BL/6 males were UV induced for 32 min as described above until the appearance of skin lesions and randomly assigned to three groups: (1) Skin smeared with 200 μL PBS, (2) Skin smeared with 200 μL ADMSC-EXOs (1 ug/μL), (3) Skin ameared with 200 μL si-ADMSC-EXOs. Treatments were given 3 times per week for 2 weeks.

### H&E staining

Mice were euthanized and UV irradiated and control skin were surgically removed, fixed using 4% paraformaldehyde. Skin tissues were embedded in paraffin, sectioned (8 μM thick), UV induced inflammation, cellular infiltration and skin damage were assessed with H & E staining.

### ELISA

The cell supernatant and mouse skin tissues were collected and centrifuged at 1000 g for 10 min, followed by 10000 g for 30 min, and the supernatant was taken to a clean EP tube. The levels of cytokines in the samples were then analyzed using an inflammatory cytokine multi analyte ELISArray Kit (Qiagen, Germany) according to the manufacturer’s instructions. Absorbance (450 nm) was measured using a multifunctional microplate reader.

### Statistical analysis

All values are expressed as mean ± SEM or mean ± SD. Statistically significant differences between the groups were analyzed using one-way or two-way analysis of variance (ANOVA). Differences with *P* < 0.05 were considered statistically significant. (Statistical significance was designated as ^*^ *P* < 0.05, ^**^ *P* < 0.01, ^***^ *P* < 0.005, and ^****^ *P* < 0.001).

## Results

### ADMSC derived exosomes directly taken up by skin

**Figure 1:**
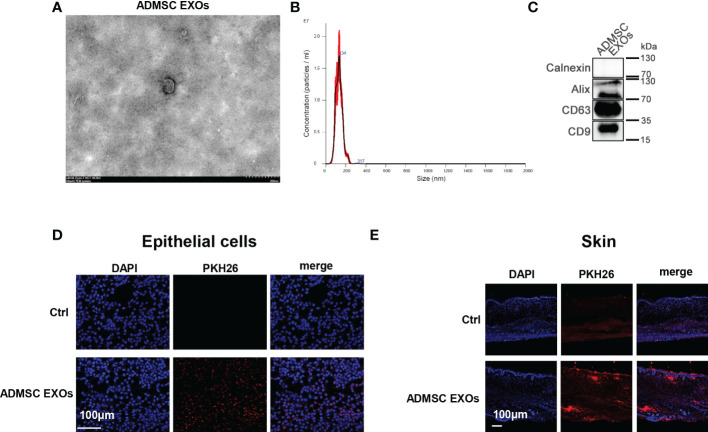
ADMSC derived exosomes directly taken up by skin. **(A, B)** Transmission electron microscopy **(A)** and nanoparticle size tracking **(B)** assay of exosomes extracted from ADMSC cell culture medium. **(C)** Analysis of the protein markers (CD9, CD63, and Alix) in ADMSC exosomes through western blot. **(D, E)** PKH26 stained ADMSC exosomes could be taken up by epithelial cells **(D)** and mouse skin tissues **(E)**.

Exosomal membrane is a kind of cell membrane like structure, and exosomes can be taken up by cells or tissues in the way of membrane fusion. To explore the contribution of skin uptake of ADMSC derived exosomes, we characterized ADMSC exosomes obtained by ultracentrifugation and applied the stained exosomes with epithelial cells and mice skin cells for fluorescence tracing. Transmission electron microscopy (TEM) observed that exosomes extracted from ADMSC cell culture fluid were vesicle like structures with diameters ranging between 100-200 nm (**Figure 1A**); Further analysis using nanoparticle size tracking (NTA) confirmed that the size distribution of ADMSC-EXOs was between 30-200 nm, with a peak size at 130 nm and a concentration of approximately 9×10^11^ particles/ml (**Figure 1B**). We next performed western blot (WB) analysis of membrane proteins from ADMSC-EXOs, which express CD9, CD63, and Alix characteristic of exosomes, but not calnexin, which is part of the cell membrane skeleton (**Figure 1C**). Next, we added the PKH26 stained exosomes into culture medium of epithelial cells, and exosomes could be absorbed by epithelial cells after 4 h incubation (**Figure 1D**). Besides, the stained exosomes were applied on the surface of mouse skin tissue, and the fluorescence could be detected in cuticle and dermal tissues of skin after 4 h (**Figure 1E**). These results suggested that ADMSC derived exosomes can be taken up by epithelial cells and mouse skin, and may function as carrier for drug delivery.

### Biosynthesis of engineered si-ADMSC-EXOs

**Figure 2:**
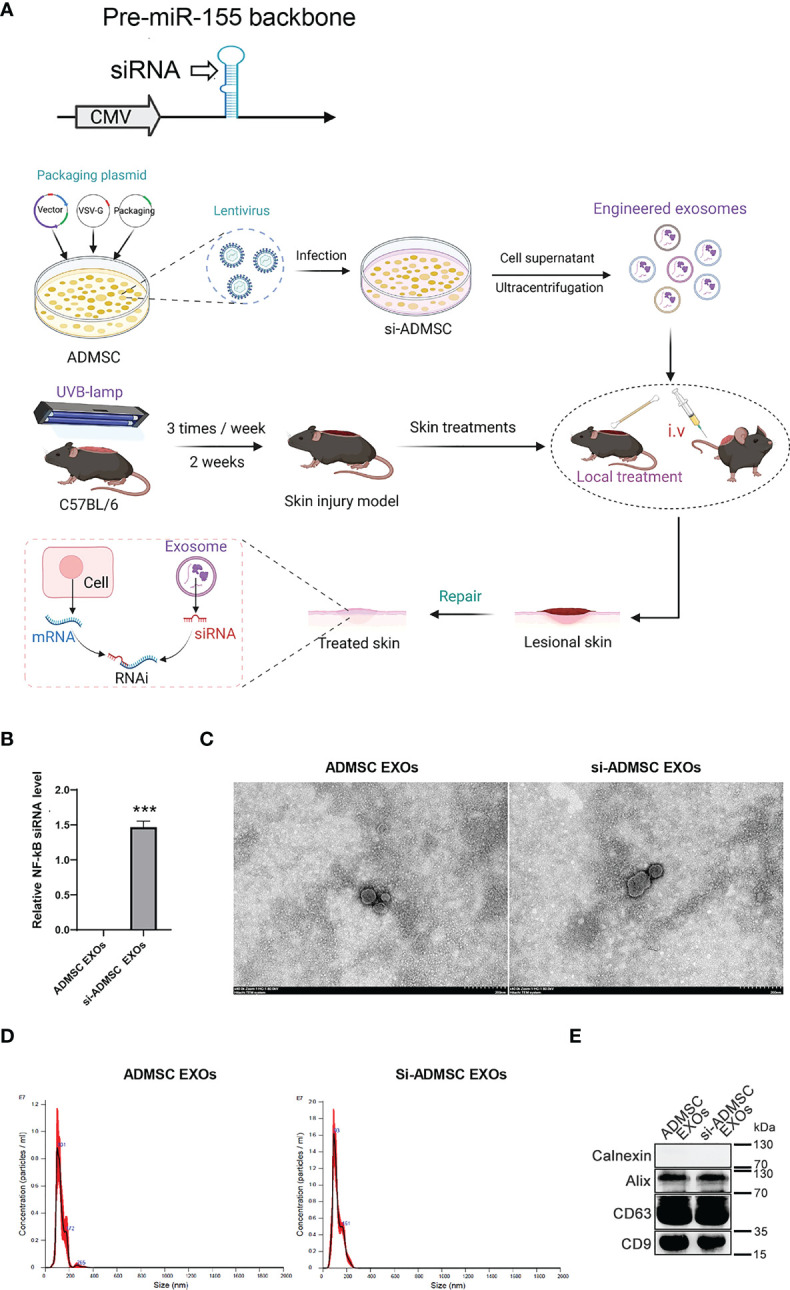
Biosynthesis of engineered si-ADMSC-EXOs. **(A)** Schematic of the experimental design. Genetic circuit were constructed consisting of two functional modules: the promoter module drives the transcription of siRNA, which leads to the package of saturated cytoplasmic siRNA into exosomes, while the siRNA expression cassette module maximises the expression of the siRNA guide strand and minimises the expression of undesired passenger strand. The cytomegalovirus (CMV) promoter was selected as the promoter module, and the pre-miR-155 backbone was selected as the optimal siRNA expression cassette to produce siRNA. We then constructed lentivirus containing these siRNA-expressed genetic circuit and infected with ADMSC cells. siRNA expressed-ADMSC cells were selected (si-ADMSCs) and the secreted exosomes were obtained through ultracentrifugation. The skin lesions were treated through swearing the si-ADMSC exosomes. **(B)** Quantitative PCR analysis the siRNA level of NF-κB in si-ADMSC exosomes and normal ADMSC exosomes. **(C-E)** Transmission electron microscopy **(C)**, nanoparticle size tracking **(D)**, western blot **(E)** assay of exosomes extracted from si-ADMSC and ADMSC cell culture medium. ***p<0.001.

The therapeutic use of siRNA is limited by the inability of RNA molecules to reach their target cells. Recently, nature’s own carriers of RNA, exosomes, are increasingly being considered as siRNA delivery vehicles due to their properties. NF-κB (nuclear factor kappa light chain enhancer of activated B cells), which is one of the key regulators of inflammatory immune responses and regulated cytokine production (e.g., interleukin-6 (IL-6) and TNF-α), plays an integral role in the pathogenesis of skin lesions. We therefore evaluated the therapeutic effects of the siRNA designed to specifically target NF-κB for the treatment of skin lesions. We constructed a genetic circuit consisting of two functional modules: the promoter module drives the transcription of siRNA, which leads to the package of saturated cytoplasmic siRNA into exosomes, while the siRNA expression cassette module maximises the expression of the siRNA guide strand and minimises the expression of undesired passenger strand. Besides, the cytomegalovirus (CMV) promoter was selected as the promoter module, and the pre-miR-155 backbone was selected as the optimal siRNA expression cassette to produce siRNA. To make the siRNA of target genes highly expressed in ADMSC exosomes, we constructed lentivirus containing these siRNA-expressed genetic circuit. After the lentivirus infected the cell, we could select the siRNA stablely expressed ADMSC cell line (si-ADMSCs). The si-ADMSC exosomes were then obtained by ultracentrifugation (**Figure 2A**). Quantitative PCR revealed that the siRNA expression of NF-κB was significantly higher in si-ADMSC exosomes (si-ADMSC-EXOs) than in normal ADMSC-EXOs (**Figure 2B**). TEM results confirmed that there was no obvious difference in the morphology between si-ADMSC-EXOs and ADMSC-EXOs, and NTA analysis also indicated that there were no significant differences in particle number and size distribution between the two types of exosomes (**Figures 2C, D**). Proteins of exosomes were also detected by WB, and the amount of protein expression of si-ADMSC-EXOs was similar with that of ADMSC-EXOs (**Figure 2E**). These results suggested the successful synthesis of siRNA overexpressed si-ADMSC-EXOs, and the characterization of si-ADMSC-EXOs and ADMSC-EXOs was not significantly different, providing the potential for gene therapy in skin lesions.

### Evaluation of the activity of NF-κB siRNA-encapsulating exosomes *in vitro*

**Figure 3:**
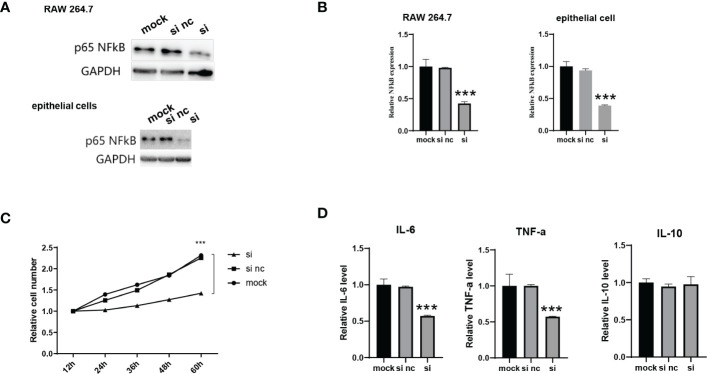
Evaluation of the activity of NF-κB siRNA-encapsulating exosomes *in vitro*. **(A, B)** The NF-κB protein **(A)** and mRNA **(B)** level of primary macrophages cells (RAW 264.7) and epithelial cells after incubating with si-ADMSC-EXOs (si) and nc-ADMSC-EXOs (si nc). **(C)** CCK-8 assay of RAW 264.7 cells after incubating with si-ADMSC-EXOs (si) and nc-ADMSC-EXOs (si nc). **(D)** ELISA assay of IL-6, TNF-α and IL-10 level in RAW 264.7 cells after incubating with si-ADMSC-EXOs (si) and nc-ADMSC-EXOs (si nc). ***p<0.001.

We evaluated whether the formation of NF-κB siRNA-encapsulating exosomes could reduce NF-κB expression *in vitro*. Macrophages play a central role in all stages of wound healing and orchestrate the wound healing process. Their functional phenotype is dependent on the wound microenvironment, which changes during healing, hereby altering macrophage phenotype. Pathological functioning of macrophages in the wound healing process can result in derailed wound healing, like the formation of ulcers, chronic wounds, hypertrophic scars and keloids. We first incubated si-ADMSC-EXOs with macrophage cells (RAW 264.7 cell lines) and epithelial cells, respectively, and detected the expression levels of the NF-κB. Significant decrease in NF-κB protein and mRNA expression was observed in both primary macrophages and epithelial cells (**Figures 3A, B**). We then explored the function of NF-κB siRNA-encapsulating exosomes in macrophage proliferation by CCK-8 assay. Compared with the mock or control group, the engineered si-ADMSC-EXOs significantly inhibited the proliferation of RAW 264.7 cells (**Figure 3C**). Next, we measured the levels of cytokines in RAW 264.7 cells through ELISA, and we found that the expression levels of IL-6 and TNF-α, but not IL-10, were decreased in RAW 264.7 cell relative to controls (**Figure 3D**). These results indicated that the biosynthesized engineered si-ADMSC-EXOs can effectively inhibit the expression of the target genes and inhibit the macrophage activation.

### *In vivo* therapeutic effects of engineered si-ADMSC-EXOs in skin injury mice model

**Figure 4:**
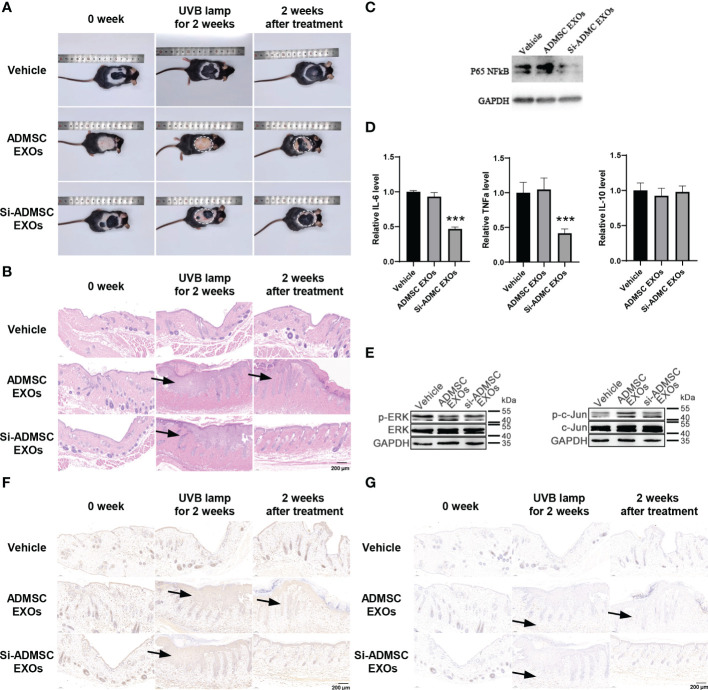
*In vivo* therapeutic effects of engineered si-ADMSC-EXOs in skin injury mice model. **(A)** Representative macroscopic features of skin injury. The skin injury model in C57BL/6 mice were constructed by two weeks of UV irradiation and then treated by smearing ADMSC-EXOs or si-ADMSC-EXOs exosomes; while control mice were set without UV irradiation (vehicle). **(B)** Representative images of H&E staining of mice skin injury tissues. **(C)** The protein level of NF-κB in the treated skin tissues. **(D)** ELISA assay of IL-6, TNF-α and IL-10 level in the treated skin tissues. **(E)** The protein level of phosphorylated ERK protein (p-ERK) and c-Jun protein (p-c-Jun) in the treated skin tissues. **(F, G)** Immunohistochemical analysis of the macrophage markers (F480 and CD206) in the treated skin tissues. ***p<0.001.

Above, we confirmed that si-ADMSC-EXOs could be taken up by epithelial cells and skin, while suppressing the expression of target genes and inhibiting the proliferation and cytokines of macrophages *in vitro*. Here, we constructed skin injury model in C57BL/6 mice by two weeks of UV irradiation and then treated by smearing ADMSC-EXOs or si-ADMSC-EXOs, respectively (**Figure 2A**), while negative control mice were treated without UV irradiation (vehicle). Comparing with vehicle group mice, two weeks of UV irradiation caused the mice acute skin redness and ulceration in both ADMSC-EXOs and si-ADMSC-EXOs group (**Figure 4A**). As expected, treating with si-ADMSC-EXOs could faster recovery the injury in skin comparing with ADMSC-EXOs group (**Figure 4A**). H&E staining was further employed to observe the skin pathological changes, and UV irradiation would change the nuclei of skin tissue into irregular morphology, and the epidermal layer became thicker, while the si-ADMSC-EXOs treatment group showed a higher rate of skin recovery than ADMSC-EXOs group (**Figure 4B**). WB analysis of the treated skin revealed that the expression of NF-κB was no significantly different between the ADMSC-EXOs and vehicle groups, but remarkably reduced in the si-ADMSC-EXOs group (**Figure 4C**). Consistently, the expression levels of IL-6 and TNF-α, but not IL-10 were decreased in si-ADMSC-EXOs group comparing to ADMSC-EXOs group (**Figure 4D**). Besides, the level of phosphorylated ERK protein (p-ERK) and c-Jun protein (p-c-Jun) were lower in si-ADMSC-EXOs group, indicating the skin inflammation was reduced (**Figure 4E**). Finally, by immunohistochemical analysis, the level of the macrophage marker F480 in the skin tissue was higher in the UV irradiated group than in the control group, whereas the level of the M2 type macrophage marker CD206 was higher after treatment with si-ADMSC-EXOs than in the other two groups (**Figures 4F, G**). These results suggested that UV irradiation promoted an increase in M1 macrophages in the skin, which secrete inflammatory cytokines; while si-ADMSC-EXOs treatment could ultimately induce M2 type macrophage activation and inhibit inflammatory responses, thereby repairing and remodeling damaged skin cells.

## Discussion

Despite recent advancements in our knowledge of the pathogenic mechanisms of skin lesions, considerable unmet medical methods were needed for the repair of skin lesions for patients after UV light and chemotherapy drugs. Ectopic immune responses are one of the most promising therapeutic targets, and therapy using cytokines (e.g., TGF-β) has been specifically developed to alleviate immune responses (20). However, the weak therapeutic effect, high treatment costs and serious side effects remain serious problems for biological therapy. Therefore, it is urgent to develop an alternative strategy with high therapeutic efficiency and few side effects. Because siRNA is solely dependent on the mRNA sequence and inhibits immunological targets with strong specificity, RNAi therapy has the intrinsic ability to overcome the shortcomings of biological therapy (21). Unfortunately, the lack of safe and effective carriers for the delivery of siRNA therapeutics remains a major problem to its clinical application. For skin delivery, an ideal siRNA carrier must overcome a series of biological hurdles: it should protect siRNA from degradation by RNases in the skin surface, have proper permeability of the epidermis and no stimulatory effects on immune cells.

RNA interference is a sequence-specific, post-transcriptional gene silencing mechanism in animals and plants, offering an opportunity to inhibit mRNAs and modulate the expression of corresponding proteins, and RNAi have therefore greatly enlarged the proportion of human proteins that can be therapeutically manipulated (22, 23). The major challenge for RNAi-based therapy is the lack of an efficient *in vivo* delivery system, had elicited tremendous efforts to overcome this hurdle, including using delivery vehicles and conjugated ligands (24). In recent years, exosomes have emerged as potential drug carrier for RNA or protein, which can effectively delivery drug to recipient cells (25). Owing to their low immunogenicity, allogenic MSC- exosomes are available for repeated administration to patients without substantial side effects (25). Zhang et al. recently reported that hypoxic microenvironment induced microRNA-125b were packaged into MSC-derived exosomes, and exosomes can be taken up by endothelial cells and accelerate wound healing (11). This study demonstrated the superiority of the engineered exosomes platform for the delivery of siRNA/miRNA to targeted cell populations *in vivo*. We used a similar concept of self-assembly based on the nature of exosomes because exosomes and their cargoes are self-assembled by cells. Based on the intrinsic ability of the small RNA processing machinery of the MSCs to self-assemble siRNA into exosomes, we designed genetic circuits to engineer MSCs to self-assemble siRNAs into exosomes. Considering the short in cell half-life of the genetic circuits formed as naked DNA plasmids, we selected lentivirus as the carrier of genetic circuits because lentivirus is capable of establishing long-term transgene expression in adipose mesenchymal stem cells. Then we cultured the engineered adipose mesenchymal stem cells and collected the culture medium. We isolated the exosomes (*si-ADMSC-EXOs*) and daubed them to the sites of wounding, which resulted in significant target gene reduction and symptom alleviation in acute wounding models.

The molecular mechanism underlying the self-assembly of siRNAs into exosomes is not known. Recent studies on secretory miRNAs may provide some inspiration for this question. There are several evidences that cells selectively package certain miRNAs into EVs for active secretion (26). The promoter, miRNA/siRNA sequence and pre-miRNA structure may join together to decide the sorting route of siRNAs to exosomes in the genetic circuit design. According to previous reports, we selected CMV promoter and pre-miR-155 structure to express siRNA in MSC cells, and the siRNA were found enriched in the exosomes (26). However, there is much room for optimizing the structure of genetic circuits to guarantee the preferential sorting of siRNAs into exosomes rather than retaining them within cells.

Taken together, this study established a convenient, effective and safe strategy for assembling and delivery of siRNAs in mouse models of skin lesions. This technology has important theoretical significance and translational value because it may provide a significant therapeutic benefit for skin lesions.

## Data availability statement

The raw data supporting the conclusions of this article will be made available by the authors, without undue reservation.

## Ethics statement

The animal study was reviewed and approved by Laboratory Animal Ethics Committee of Wenzhou Medical University.

## Author contributions

These authors were involved with this manuscript: WL and XL (study concept and design, analysis and interpretation of data); WL (drafting of the manuscript); WL, XL, YW, JZ and WS (acquisition of data; analysis and interpretation of data; statistical analysis); WL, WS and ZJ (technical or material support). All authors contributed to the article and approved the submitted version.
